# Systemic antibiotics cause deterioration of emphysema associated with exaggerated inflammation and autophagy

**DOI:** 10.1038/s12276-023-01099-6

**Published:** 2023-10-02

**Authors:** Na Hyun Kim, Bo-Yun Choi, Eun Sil Kim, Su Jung Kim, Jeong Yeon Hong, Sun-Hee Heo, Jin-Yong Jeong, Kyunggon Kim, Hyun Ju Yoo, Woo Jun Sul, Sei Won Lee

**Affiliations:** 1 Department of Pulmonary and Critical Care Medicine, Asan Medical Center, University of Ulsan College of Medicine, Seoul, Republic of Korea; 2 Department of Systems Biotechnology, Chung-Ang University, Anseong, Gyeonggi-do Republic of Korea; 3 Department of Convergence Medicine, Asan Institute for Life Sciences, Asan Medical Center and Department of Microbiology, University of Ulsan College of Medicine, Seoul, Republic of Korea; 4 Department of Convergence Medicine, Asan Medical Center, Department of Digital Medicine, University of Ulsan, College of Medicine, Seoul, Republic of Korea

**Keywords:** Respiratory tract diseases, Cell death and immune response, Innate immunity

## Abstract

The interaction between the microbial environment and the host is important for immune homeostasis. Recent research suggests that microbiota dysbiosis can be involved in respiratory diseases. Emphysema is a chronic inflammatory disease, but it is unclear whether dysbiosis caused by antibiotics can affect disease progression. Here, we tried to elucidate the effect of systemic antibiotics on smoking-exposed emphysema models. In this study, the antibiotic mixture caused more alveolar destruction and airspace expansion in the smoking group than in the smoking only or control groups. This emphysema aggravation as a result of antibiotic exposure was associated with increased levels of inflammatory cells, IL-6, IFNγ and protein concentrations in bronchoalveolar lavage fluid. Proteomics analysis indicated that autophagy could be involved in antibiotic-associated emphysema aggravation, and increased protein levels of LC3B, atg3, and atg7 were identified by Western blotting. In microbiome and metabolome analyses, the composition of the gut microbiota was different with smoking and antibiotic exposure, and the levels of short-chain fatty acids (SCFAs), including acetate and propionate, were reduced by antibiotic exposure. SCFA administration restored emphysema development with reduced inflammatory cells, IL-6, and IFNγ and decreased LC3B, atg3, and atg7 levels. In conclusion, antibiotics can aggravate emphysema, and inflammation and autophagy may be associated with this aggravation. This study provides important insight into the systemic impact of microbial dysbiosis and the therapeutic potential of utilizing the gut microbiota in emphysema.

## Gut microbiota: the unexpected player in emphysema development

Antibiotics may aggravate emphysema by disrupting gut microbiota, according to a study on mice. Researchers found that a mixture of antibiotics led to increased alveolar destruction and inflammation in smoking-exposed emphysema models. However, short-chain fatty acids (SCFAs), which are metabolites of gut microbiota, were found to attenuate emphysema development. The study suggests that reasonable prescription of antibiotics and therapeutic potential of gut microbiota restoration and its metabolites could be beneficial for chronic respiratory diseases like emphysema.

## Introduction

Smoking causes various chronic respiratory diseases, including chronic obstructive pulmonary disease (COPD), idiopathic pulmonary fibrosis, chronic bronchitis, asthma and lung cancer^1^. Among them, COPD is a chronic progressive disease with global mortality associated with the inflammatory response and lung injury caused by exposure to noxious particles^2^, and smoking represents the greatest risk factor. COPD is a heterogeneous disease; many patients have never smoked, and only some heavy smokers have airflow limitation^3,4^. This heterogeneity has been explained by environmental and genetic factors^5^, which cannot explain every aspect. Immune homeostasis can also be involved because it is crucial in smoking susceptibility, and recent reports have suggested that microorganisms regulate immune homeostasis^6,7^.

Indeed, the association between respiratory diseases and the microbiota was suggested epidemiologically even when the concept of the microbiome was still in its infancy^8^. Now, the systemic effect of the intestinal microbiome has become relatively definitive on the brain, liver, and lung beyond the gut. This notion has led to the coining of a new, now established term, the gut–lung axis, explaining the close relationship between an imbalance in intestinal microorganisms and the pathogenesis of lung diseases^9–11^. This relationship suggests that the manipulation of the microbiota can also be applied to the treatment of respiratory diseases. Recent studies have shown that the modulation of the intestinal microbiota by diet and fecal microbiota transplantation (FMT) attenuated emphysema development, indicating the therapeutic potential of a healthy microbiota on COPD utilizing the concept of the gut–lung axis^12,13^.

Antibiotics are widely prescribed in the acute exacerbation of respiratory diseases^14^. Selective and narrow-spectrum antibiotics can be applied in some cases, but broad-spectrum antibiotics are inevitable in most clinical situations because causative organisms are not definite at initial presentation. In addition to antibiotic resistance and unnecessary side effects due to their overuse^15^, it should also be acknowledged that antibiotics are the representative treatment causing an imbalance in the intestinal microbiota. It has already been documented that the imbalance and depletion of microbiota can aggravate pulmonary infection from both bacteria^16^ and viruses^17^. Moreover, the potential effects of antibiotics on COPD from this view of microbiome imbalance have not been adequately addressed. Here, we analyzed whether antibiotic treatment can aggravate emphysema utilizing a smoking-exposed murine model. Additionally, we addressed whether this aggravation can be improved with bacterial metabolites.

## Materials and methods

### Emphysema mouse model

Seven-week-old inbred female C57BL/6 mice (Orient Bio, Seongnam, Korea) were maintained at room temperature with a 12-hour light:dark cycle. The mice were exposed to cigarette smoke 7 days a week for 4 weeks. Cigarette smoke exposure per day (4 cigarettes/session, 3 sessions/day, 8.0 mg of tar/cigarette, and 0.70 mg of nicotine/cigarette, Camel) was conducted in accordance with a previously described protocol with modifications. The control animals inhaled only clean-room air from the cages.

### Antibiotic treatment

To induce depletion of the gut microbiota, C57BL/6 mice were treated with a broad-spectrum antibiotic cocktail consisting of ampicillin (1 g/L, Sigma, St. Louis, MO, USA), vancomycin (0.5 g/L, Sigma), metronidazole (1 g/L, Sigma) and enrofloxacin (0.27 g/L, Sigma) in the drinking water ad libitum (200 ml per cage) for 3 weeks before the initiation of the smoking experiments. Drinking water was replaced once every 3 days.

### Separation and preparation of samples

After 3 weeks, the animals were anesthetized using isoflurane inhalation, and blood samples were collected by heart puncture. Feces were harvested during the final sampling process. The trachea was catheterized and perfused with 1.5 mL of PBS. The cellular and liquid fractions of the bronchoalveolar lavage fluid (BALF) were separated by centrifugation at 2200 rpm for 10 min at 4 °C. The cell pellet was suspended in PBS, attached to a glass slide and stained with Diff-Quick (Sysmex, Kobe, Japan). After ligating the bronchus by separating the left lobe from the rest of the lungs, the left lung lobe was inflated with 0.5% low-melting-point agar at a constant pressure of 15 cm H_2_O. For histological examination, the left lung lobe was sectioned and fixed in 10% formaldehyde. All specimens were collected, fixed, immediately frozen, and stored at −80 °C for analysis.

### Histomorphological assessment

Paraffin-embedded lung lobe section (5 µm) were prepared and stained with hematoxylin and eosin (H&E). The MLI, which is a measurement of the mean interalveolar septal wall distance determined by the number of interruptions in 1-mm lines of the alveolar wall, was used to assess emphysematous changes. Four lines were drawn in each field, and at least five different fields were examined in each mouse.

### Quantification of cytokine levels

The levels of IFNγ and IL-6 in the BALF were measured using a commercially available ELISA kit (R&D Systems, Minneapolis, MN, USA) following the manufacturer’s instructions.

### Quantitative real-time PCR analysis

Total RNA was extracted from liver tissue using TRIzol reagent (Thermo Fisher Scientific, Waltham, MA, USA), as directed by the manufacturer. cDNA was synthesized from the M-MLV Reverse Transcriptase buffer pack (Promega, Madison, WA, USA), dNTP Mixture (2.5 mM) (Takara, Kyoto, Japan), and random primers (20 µg) (Promega). Transcript levels were measured using real-time polymerase chain reaction (PCR) with sequence-specific primers for TNFα, IL-6, IFNγ and p53. Amplification reactions were carried out in a Light Cycler 480 System (Roche, Mannheim, Germany) using GoTaq® qPCR Master Mix (Promega) according to the manufacturer’s instructions. The target gene expression levels were normalized to 18 S RNA as an endogenous control gene. The equation $2^{\hbox{-}{\Delta\Delta}{\rm{CT}}}$ was used to calculate the relative changes.

### Peptide sample preparation

Each BAL sample was dried and dissolved in 100 μL of 5% sodium dodecyl sulfate in 50 mM triethylammonium bicarbonate (pH 8.5). After adding 20 mM dithiothreitol, the samples were incubated at 95 °C for 10 min, and the reduced sample was placed in 40 mM iodoacetamide and incubated for 30 min at room temperature in the dark. With a final concentration of 1.2% phosphoric acid, samples were attached to S-Trap mini columns (ProtiFi, Farmingdale, NY, USA; Cat. No: CO2-mini-80). Following the manufacturer’s protocol, we performed suspension-trapping (S-trap) proteolysis by adding 4 μg of Lys-C/trypsin mixture at 37 °C for 16 h^18^. The digested peptide mixture was desalted using reversed-phase chromatography with C18 chemistry, and the dried peptide samples were stored at −80 °C until use.

### nanoLC‒MS/MS

Peptides were separated using the Dionex UltiMate 3000 RSLCnano system (Thermo Fisher Scientific). Each dried sample was reconstituted with 25 µL of 0.1% formic acid, a 5-µL aliquot of which was injected into a C18 Pepmap trap column (20 mm × 100 µm i.d., 5 µm, 100 Å; Thermo Fisher Scientific) and separated by an Acclaim™ Pepmap 100 C18 column (500 mm × 75 µm i.d., 3 µm, 100 Å; Thermo Fisher Scientific) over 200 minutes (250 nL/min) using a 0% to 48% acetonitrile gradient in 0.1% formic acid and 5% DMSO for 150 minutes at 50 °C. The liquid chromatography column was coupled to a Q Exactive mass spectrometer (Thermo Fisher Scientific) with a nano-ESI source. Mass spectra were acquired in data-dependent mode with an automatic switch between a full scan and 20 data-dependent MS/MS scans. The target value for the full scan MS spectra was 3,000,000 with a maximum injection time of 100 ms and a resolution of 70,000 at *m/z* 400. The ion target for MS/MS was set to 1,000,000 with a maximum injection time of 50 ms and a resolution of 17,500 at *m/z* 400. Repeated peptides were dynamically excluded for 20 s.

### Proteomic identification and quantification

The acquired MS/MS spectra were retrieved on the SequestHT on Proteome Discoverer (version 2.4; Thermo Fisher Scientific) and compared with the SwissProt human protein sequence database (March 2021)^19^. The precursor mass tolerance was set to ±10 ppm, and the MS/MS tolerance was set at 0.02 Da. The search parameters were set to default parameters, including cysteine carbamidomethylation as a fixed modification and N-terminal acetylation, methionine oxidation and phospho-serine, -threonine, and -tyrosine as variable modifications with two miscleavages. False discovery rates were set at 1% for each analysis using “Percolator”^20^. From the Sequest search output, peptide filters that included peptide confidence, peptide rank, score versus charge state, and search engine rank were set at the default values for Proteome Discoverer. In global proteome analysis, proteins were quantified label free using the peak intensity for unique and razor peptides of each protein and excluded peptides that involved methionine oxidation.

### GO analysis and statistical analysis

Pathway enrichment analysis was performed by ShinyGO (version 0.76.1, http://bioinformatics.sdstate.edu/go/). Principal component analysis, volcano plot analysis and Venn diagram analysis were performed in Proteome Discoverer (ver 2.4; Thermo Fisher Scientific).

### Quantitative short-chain fatty acid (SCFA) measurement

Standard metabolites and internal standards were purchased from Sigma‒Aldrich. Ten to twenty milligrams of feces was freeze-dried for 12 h using a Benchtop manifold freeze drier and stored at −80 °C with harvested serum until analysis. For sample preparation, the fecal sample was vortexed vigorously with 150 μL of internal standard solution (1 mM propionic acid (C3)-d6) in water and centrifuged at 13,200 rpm for 10 min at 4 °C. The supernatant was then collected. For mouse serum analysis, 20 μL of serum was mixed with 150 μL of internal standard solution (1 mM propionic acid (C3)-d6) in water and mixed well. The solution was centrifuged at 13,200 rpm for 10 min at 4 °C, and the supernatant was collected. Then, 100 μL of 20 mM AABD-SH in dichloromethane, 100 μL of 20 mM TPP in acetonitrile, and 100 μL of 20 mM DPDS in acetonitrile were added to the supernatant. The solution was incubated for 10 min at room temperature while vortexing and dried under vacuum. The dried matter was reconstituted with 20 μL of methanol to prepare for liquid chromatography–tandem mass spectrometry (LC–MS/MS) analysis. An LC–MS/MS system equipped with 1290 HPLC (Agilent Technologies, Glostrup, Denmark), Qtrap 5500 (ABSciex, Framingham, MA), and a reverse-phase column (Pursuit 5 C18 150 × 2.0 mm, Agilent Technologies) was used. MS was operated in the positive ion mode with a turbo ion-spray voltage of 5500 V using 20 psi curtain gas, 50 psi nebulizer gas, and 50 psi drying gas at 400 °C. The LC separation used mobile phase A (0.1% formic acid in water) and mobile phase B (0.1% formic acid in acetonitrile) and proceeded at 500 µl/min and 40 °C. The separation gradient was as follows: 30% B at 0 min, 50% B for 30 min, 50–30% B for 0.1 min, and 30% B for 4.9 min. Collision energies of 15 V were used for multiple-reaction monitoring (MRM) of each SCFA. LC–MS/MS data were analyzed with Analyst 1.5.2 software (ABSciex). The extracted ion chromatogram (EIC) corresponding to the specific transition for each metabolite was used for quantitation. The area under the curve of each EIC was normalized to the EIC of the internal standard. The peak area ratio of each metabolite to the internal standard was normalized using the serum volume of a sample before being used for relative comparison. The internal standard for mouse feces was not detected; thus, the results are presented as the analyte peak area.

### Short-chain fatty acid administration

The SCFAs acetate and propionate (Sigma‒Aldrich, St. Louis, MO, USA) were administered intraperitoneally (IP) and intrarectally (IR) at 1 g/kg twice a week for five weeks during the experiment and lso in drinking water at 200 mM throughout the 5 weeks of the experimental period.

### DNA extraction and bacterial 16 S rRNA gene sequencing

Total genomic DNA from fecal samples was extracted using a FastPrep-24 instrument (Qbiogene, MP Biomedicals, Illkirch, France) and processed according to the manufacturer’s instructions. The V4 region of the 16 S rRNA gene was amplified using 515 F and 806 R primers designed for dual indexing and pooled, and the Illumina iSeq100 platform was used for the analysis.

### Microbiome data analysis

We merged OTU tables of each sample using R and rarefied them with a depth of 9370 sequences per sample. We used the QIIME^TM^ (Quantitative Insights Into Microbial Ecology) 2 pipeline^21^ to conduct the microbial diversity analyses. Beta diversity was calculated using the QIIME diversity beta plugin, and principal coordinates analysis (PcoA) based on Jaccard and Bray‒Curtis distances calculated using the QIIME diversity pcoa plugin was used to verify the microbial compositional differences between groups. We performed linear discriminant analysis effect size (LefSe)^22^ to identify differential taxonomy between groups. To compare all biomarkers among groups, the Kruskal‒Wallis rank-sum test was used in LefSe. We performed the Wilcoxon rank-sum test in R to identify significant differences in dissimilarities.

### Western blot analysis

Lung tissue samples were homogenized in protein lysis buffer (200 mM PMSF, 1 mg/ml leupeptin, 1 mg/ml aprotinin, 200 mM Na3VO4, 200 mM NaF). After centrifugation for 30 min at 13,000 rpm, the supernatants were recovered. Typically, 20 μg of protein per lane was loaded on SDS‒PAGE gels and transferred onto nitrocellulose membranes. The membranes were blocked for 1 h with 5% skim milk in Tris-buffered saline containing 0.05% Tween 20 (TBST) and were incubated overnight at 4 °C with primary antibodies against LC3B(I,II), ATG3, ATG5, ATG7 and GAPDH. After washing with TBST, the membranes were incubated for 1 h with HRP-conjugated secondary antibodies and developed using chemiluminescence HRP substrates.

## Results

### Antibiotic administration aggravated the murine emphysema model

**Fig. 1:**
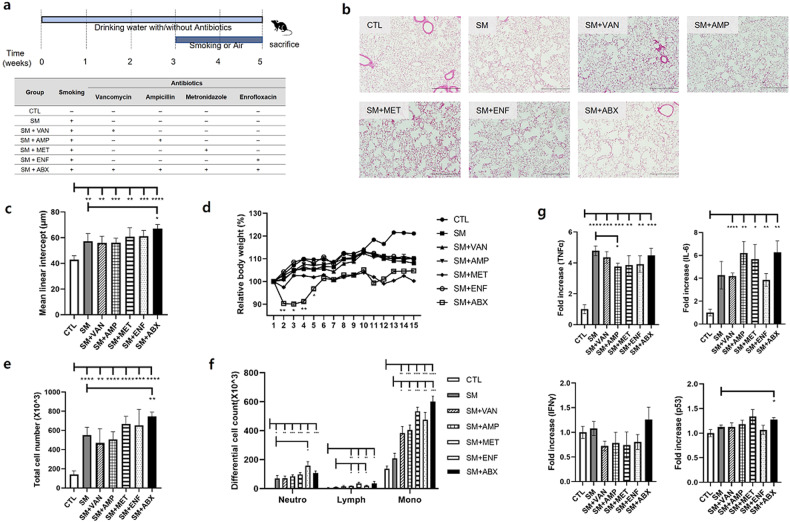
The effect of each antibiotic on the emphysema model. **a** First experimental design. **b** Representative H&E-stained lung tissues from mice in the control, emphysema, and emphysema with vancomycin, ampicillin, metronidazole, enrofloxacin, and antibiotic mixture groups (magnification: ×100). **c** The mean linear intercept (MLI) of lung tissues from each group. **d** Relative body weight during the 5 weeks of the experimental period. **e** Total number of cells in the BALF infiltrating the airways. **f** Differential cell numbers in the BALF in each group. **g** The relative mRNA levels of TNFα, IL-6, IFNγ and p53 in lung tissues (*n* = 4 control mice, *n* = 5 emphysema mice, *n* = 5 emphysema with vancomycin mice, *n* = 5 emphysema with ampicillin mice, *n* = 5 emphysema with metronidazole mice, *n* = 5 emphysema with enrofloxacin mice, *n* = 4 emphysema with antibiotic mixture mice). Values are expressed as the mean ± SEM. *P < 0.05, **P < 0.01, and ***P < 0.001. CTL control, SM smoking, SM + VAN vancomycin treatment, SM + AMP ampicillin treatment, SM + MET metronidazole treatment, SM + ENF enrofloxacin treatment, SM + ABX antibiotic mixture.

To determine whether antibiotic administration affects emphysema, antibiotics (1 g/L ampicillin; 0.5 g/L vancomycin; 1 g/L metronidazole; 1 g/L enrofloxacin; 0.27 g/L) and their mixture were administered to the smoking-exposed emphysema model (Fig. 1a). The MLI was significantly increased with smoking exposure (SM), and the addition of each antibiotic did not increase the MLI significantly compared to the smoking exposure only group (SM). Furthermore, the antibiotic mixture with smoking (SM + ABX) caused more alveolar destruction than that in the SM group, as noted by the significantly increased MLI (Fig. 1b, c). The weight decreased at the start of smoking exposure but was gradually recovered in the SM + ABX group (Fig. 1d). With smoking exposure, inflammatory cell infiltration was increased in the BALF (Fig. 1e), and the highest count was noted in the SM + ABX group. This inflammation is distinguished by the highest macrophage infiltration among the antibiotic-treated emphysema groups (Fig. 1f). The mRNA expression of proinflammatory mediators such as tumor necrosis factor (TNF) α, IL-6, IFNγ, and the anticancer gene p53 showed some different responses to antibiotic administration (Fig. 1g). These findings suggest that antibiotic-induced microbiota imbalances exacerbated pathological changes in the lungs and influenced inflammatory responses associated with smoking exposure.

### The antibiotic mixture aggravates inflammatory markers in a mouse emphysema model

**Fig. 2:**
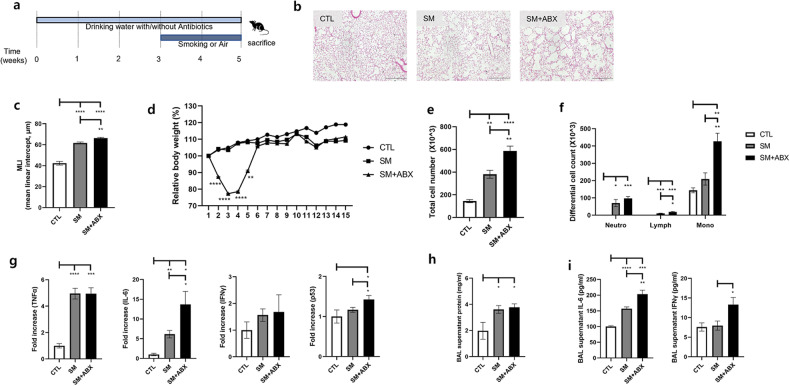
The effect of the antibiotic mixture on the emphysema model. **a** Second experimental design. **b** Representative H&E-stained lung tissues from mice in the control and emphysema with antibiotic mixture groups (magnification: ×100). The experiment was conducted in the same way as the first experiment using antibiotic cocktails. **c** The MLI of lung tissues from each group. **d** Relative body weight change during the 5 weeks of the experimental period. **e** Total number of cells in the BALF infiltrating the airways. **f** Differential cell numbers in the BALF in each group. **g** The relative mRNA levels of TNFα, IL-6, IFNγ, and p53 in lung tissues. **h** The protein level in the BALF measured using the BCA assay. **i** The levels of the cytokines IL-6 and IFNγ in the BALF were measured using ELISA. (*n* = 4 control mice, *n* = 10 emphysema mice, *n* = 10 emphysema with antibiotics mice). Values are expressed as the mean ± SEM. *P < 0.05, **P < 0.01, and ***P < 0.001. CTL control, SM smoking, SM + ABX antibiotic mixture.

Since the emphysema model was aggravated significantly by treatment with the antibiotic mixture (SM + ABX), pulmonary inflammatory responses were further analyzed in the SM + ABX group (Fig. 2a). The addition of antibiotics (SM + ABX) caused more severe lung alveolar destruction and significantly increased the MLI compared with that in the emphysema group (SM, Fig. 2b, c). The weight of the SM + ABX group decreased at the beginning of smoking, followed by a gradual recovery (Fig. 2d), similar to the previous experiment. Cell infiltration was significantly increased in the SM + ABX group, as noted by the highest total cell and macrophage numbers in the BALF (Fig. 2e, f). In addition, in the SM + ABX group, the mRNA levels of inflammatory markers, including TNFα, IL-6, and IFNγ, and the anticancer gene p53 were increased. In particular, IL-6 and p53 were significantly increased compared to levels in the SM group (Fig. 2g). The protein levels of IL-6 and IFNγ were increased in the BALF in the ABX group compared with the SM group (Fig. 2h, i). Here, we showed that an antibiotic mixture exacerbates the overall inflammatory response related to smoking-induced emphysema. Based on these results, we performed proteomics analysis to determine which mechanisms were involved when antibiotics were administered in combination with smoking exposure. Proteomics analysis revealed the difference between the SM and SM + ABX groups, and the number of increased proteins was greater in the SM + ABX group than in the SM group (Supplementary Fig. 1a, b). Each group was clearly divided in the PCA plot (Supplementary Fig. 1c), and autophagy was a dominant biological process that categorized the groups (Supplementary Fig. 1d).

### Antibiotics increase autophagy in smoking-exposed emphysema models

**Fig. 3:**
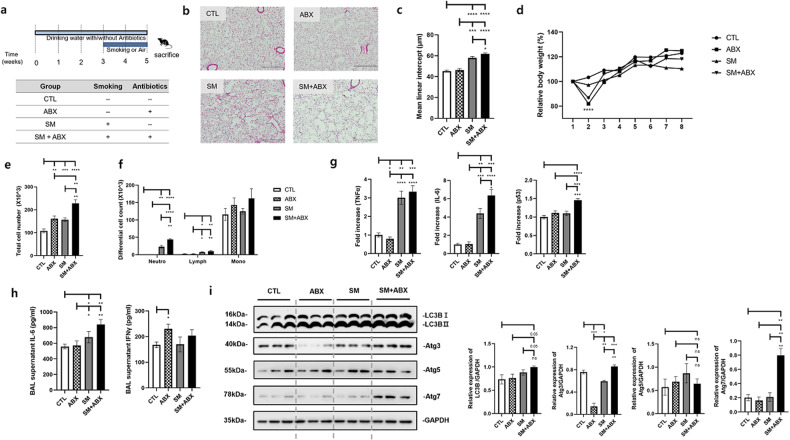
The effects of autophagy on the emphysema model with antibiotics. **a** Third experimental design. **b** Representative H&E-stained lung tissues from mice in the control, antibiotics only, emphysema and emphysema with antibiotic mixture groups (magnification: ×100). **c** The mean linear intercept (MLI) of lung tissues from each group. **d** Relative body weight during the 5 weeks of the experimental period. **e** Total number of cells in the BALF infiltrating the airways. **f** Differential cell numbers in the BALF in each group. **g** The relative mRNA levels of TNFα, IL-6, and p53 in lung tissues. **h** The levels of the cytokines IL-6 and IFNγ in the BALF measured using ELISA. **i** The expression of autophagy-related genes (LC3B, Atg3, Atg5, and Atg7) in total lung homogenates was determined by western blot analysis (*n* = 4 control mice, *n* = 7 mice treated with only antibiotics, *n* *=* 8 emphysema mice, *n* = 8 emphysema mice treated with antibiotics). Values are expressed as the mean ± SEM. *P < 0.05, **P < 0.01 and ***P < 0.001. CTL control, SM smoking, ABX antibiotics only, SM + ABX antibiotics mixture.

Antibiotic administration affected the increase in inflammatory cytokines and proteins in smoking-exposed emphysema. The following experiment was performed to determine whether autophagy-related factors were involved, as indicated by the proteomics analysis. An antibiotic-only treatment (ABX) group was included in the experimental design (Fig. 3a). Similar to the previous results, the antibiotic mixture group had the most severe alveolar destruction with an increase in the MLI. The ABX group showed an increased MLI compared with the control group but a lower or comparable level to that in the smoking exposure group (SM) (Fig. 3b, c). As in previous findings, mouse weight decreased early in the antibiotic administration group (Fig. 3d). The antibiotic mixture (SM + ABX) group showed significant cell infiltration compared to that of all other groups, and it was highest in differential cells (Fig. 3e, f). It was confirmed that the mRNA levels of inflammatory markers, including TNFα, IL-6, and p53, were increased in the SM + ABX group (Fig. 3g). Additionally, the SM + ABX group had significantly higher levels of the cytokines IL-6 and IFNγ in the BALF than the levels in the SM group (Fig. 3h). The expression levels of autophagy-related proteins were detected in lung tissue. As a result, the levels of LC3B, atg3, and atg7, excluding atg5, were high in the antibiotic mixture group. Above all, atg3 and atg7 were significantly increased compared to levels in the SM group (Fig. 3i).

### Microbial imbalance caused by the antibiotic mixture alters the gut microbiota and metabolite clusters

**Fig. 4:**
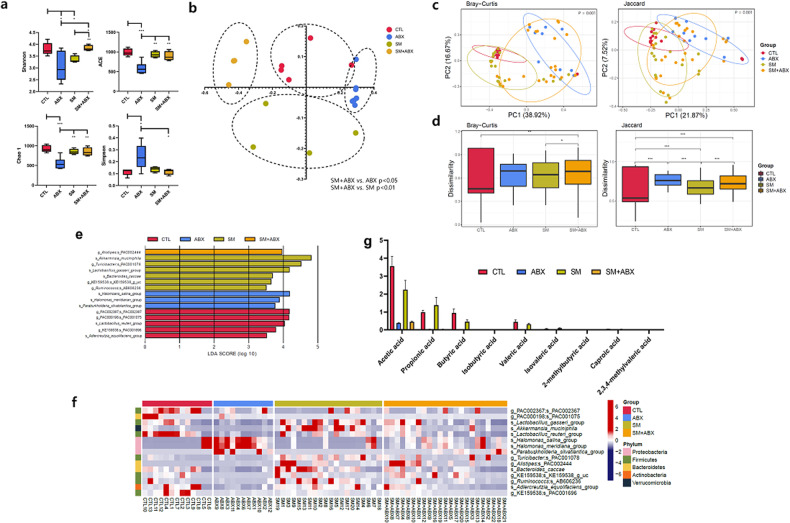
Microbial imbalance caused by the antibiotic mixture alters the gut microbiota and metabolite clusters. **a** Alpha diversity species level (UniFrac) of the four groups. **b** Beta diversity species level (UniFrac) of the four groups. **c** The PCoA results for each of the four mouse groups. **d** Dissimilarity results for each of the four mouse groups. **e** Microbiome heatmap analysis results for each of the four groups of mice. **f** Microbiome LEfSe analysis results for each of the four groups of mice. **g** SCFA metabolite analysis results for each of the four groups of mice.

It was confirmed that antibiotic administration worsened emphysema and increased autophagy. Various diversity indicators were used to distinguish changes in the gut microbiome of each group for the intestinal and lung correlation. The alpha diversity represented by the Shannon index and the abundance estimated by the Chao1 and Simpson values both showed the greatest change in alpha content in the antibiotic-treated group (Fig. 4a). Similarly, for the beta diversity, we showed separate clusters in each group (Fig. 4b).

Furthermore, a major coordinate analysis based on the relative abundance of genera revealed a distinct fecal microbial community structure. Antibiotic-induced changes in intestinal microorganisms differed significantly from other microbial community structures in the gut (Fig. 4c). There were also significant differences in nonsimilarity between the groups (Fig. 4d). Moreover, microbiome heatmap analysis and linear discrimination analysis effect size (LefSe) were used to examine changes in the microbial composition of fecal samples in each group (Fig. 4e, f). SCFA-producing bacteria such as *Lactobacillus*, *Akkermansia* and *Ruminococcus* were relatively abundant in the groups without antibiotics (control, SM), but these were depleted with antibiotic exposure. Moreover, bacteria indicating dysbiosis or contamination, such as *Alistipes* and *Halomonas*, were relatively abundant with antibiotic exposure. The local concentrations of SCFAs were also much lower in the ABX and SM + ABX groups than in the control and SM groups (Fig. 4g).

### Effect of SCFA administration on emphysema through various channels

**Fig. 5:**
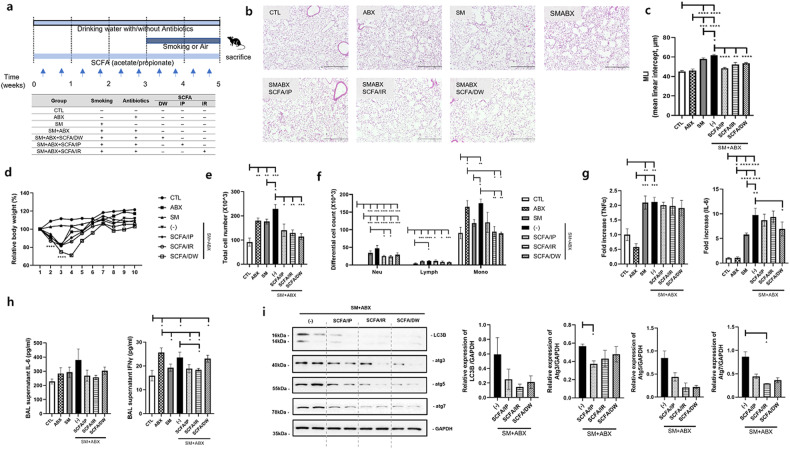
SCFA supplementation decreased the inflammatory response and emphysema. **a** First experimental design. **b** Representative H&E-stained lung tissues from mice in the control, antibiotics only, emphysema, emphysema with antibiotics mixture, and emphysema with SCFA supplementation groups (IP, IR, and DW) (magnification: ×100). **c** The mean linear intercept (MLI) of lung tissues from each group. CTL control, ABX antibiotics, SM smoking, i.p. intraperitoneal, i.r. intrarectal, DW drinking water. **d** Relative body weight during the 5 weeks of the experimental period. **e** Total number of cells in the BALF infiltrating the airways. **f** Differential cell numbers in the BALF in each group. **g** The relative mRNA levels of TNFα and IL-6 in lung tissues. **h** The levels of the cytokines IL-6 and IFNγ in BALF were measured using ELISA. **i** The expression of autophagy-related proteins (LC3B, Atg3, Atg5, and Atg7) in total lung homogenates was determined by western blot analysis (*n* = 4 control mice, *n* = 5 antibiotic-only mice, *n* = 6 emphysema mice, *n* = 8 emphysema with antibiotics mice, *n* = 6 SCFA/IP, *n* = 6 SCFA/IR, *n* = 6 SCFA/DW). Values are expressed as the mean ± SEM. *P < 0.05, **P < 0.01 and ***P < 0.001. CTL control, SM smoking, ABX only antibiotics, SM + ABX antibiotics mixture. SCFA/IP intraperitoneal injection, SCFA/IR intrarectal injection, SCFA/DW drinking water.

In a previous experiment, it was confirmed that antibiotic administration changed the microbiota and decreased SCFA levels in the gut. We hypothesized that SCFA administration could attenuate emphysema based on the beneficial effect of a high-fiber diet on emphysema associated with increased fecal SCFA concentrations^12^. SCFAs, acetic acid and propionic acid, were provided through various routes, that is, the drinking water (DW), intraperitoneal injection (IP, 3 times/week) and intrarectally (IR, three times/week), for 5 weeks (Fig. 5a) with smoking. Alveolar destruction was the most severe in the SM + ABX group, and the group with SCFA supplementation exhibited significantly decreased destruction compared to that in the SM + ABX group, which had a significantly increased MLI compared to that in the smoking group (Fig. 5b, c). Body weight was initially reduced in all groups administered smoking and antibiotics followed by recovery, as shown in previous experiments (Fig. 5d). Compared to the SM + ABX group, the total cell numbers in the BALF significantly decreased in the groups supplemented with SCFAs, especially with SCFAs administered through the IR and DW routes, and the decrease was prominent in monocytes (Fig. 5e, f). The mRNA levels of TNFα and IL-6 were decreased in the group treated with SCFAs through the DW (Fig. 5g), and IFNγ in the BALF was significantly decreased in the group treated with SCFAs IR compared to the SM + ABX group (Fig. 5h). For autophagy-related proteins in lung tissue, the group supplemented with SCFAs showed an overall decrease compared to that in the SM + ABX group, and the protein levels of LC3B and atg7 were significantly decreased in the groups supplemented with SCFAs through the IP and IR routes compared to levels in the SM + ABX group (Fig. 5i). Immunohistochemistry (IHC) staining showed that autophagy-related proteins were increased in the SM and SMABX groups compared with the CTL group, and this increased expression decreased with intraperitoneal SCFA administration (Supplementary Fig. 2a). In addition, high-resolution measurements suggested that these proteins were mainly expressed in alveolar epithelial cells and alveolar macrophages (Supplementary Fig. 2b–d).

To confirm the dose‒response relationship, SCFAs were administered intraperitoneally at three different concentrations (0.25 g/kg, 0.5 g/kg, and 1 g/kg). The MLI levels were the highest in the SM + ABX group and were lower in all SCFA-administered groups than in the SM and SM + ABX groups (Supplementary Fig. 3a). The total cell counts, neutrophils, and lymphocytes were significantly increased in the SM and SMABX groups compared with the CTL group, and they were significantly decreased in the SCFA group (1 g/kg) (Supplementary Fig. 3b, c). In addition, the mRNA levels of IL-6 and IFNγ tended to decrease as the concentration of SCFA administration increased (Supplementary Fig. 3d). All of these effects with SCFAs were the most prominent at a concentration of 1 g/kg.

## Discussion

Here, we presented the association between microbiome dysbiosis related to antibiotics and emphysema. The antibiotic mixture aggravated smoking-exposed emphysema with a change in the gut microbiota. Microbiome analysis showed a clear separation of the gut microbiome with exposure to smoking and antibiotics. Interestingly, SCFAs, the major metabolites of the gut microbiota, attenuated smoking-induced emphysema aggravation related to antibiotics. Until now, the incorporation of intestinal microbial therapy has been mostly studied in gastrointestinal or infectious diseases^23^ and several allergic diseases for respiratory diseases^24^. This study provided meaningful insight into the therapeutic potential of the microbiome and its metabolites in chronic respiratory diseases beyond the diseases that are conventionally treated by modulating microbiome. Broad-spectrum antibiotics are commonly prescribed in the treatment of COPD exacerbation, and this study suggested that excessive use of antibiotics can aggravate emphysema in the long term.

Microbiome techniques have contributed to elucidating that the lung is not a sterile organ. In addition to the local microbiota, the systemic microbiota is also closely related to lung health through immune homeostasis. The gut, as the largest reservoir of bacteria, will have the greatest impact on the lung immune environment, as supported by various studies. the commensal microbiota composition critically regulates the generation of virus-specific CD4 and CD8 T cells and antibody responses that are diminished with antibiotics^25^. Exposure of neonatal mice to commensal bacteria immediately after birth is required for a robust host defense against bacterial pneumonia and disruption of postnatal commensal colonization with the migratory program of group 3 innate lymphoid cells has negative consequences on health^26^. In specific situations, gut microbiome-induced cell migration has detrimental effects through the gut–lung axis. Gut commensal segmented filamentous bacteria trigger pathologic lung autoimmunity in the pre-arthritic phase by inducing Th17 cells^27^. This active dialog between the gut and lung is also clinically supported by the pulmonary manifestations of inflammatory bowel disease (IBD)^28^. Population-based studies have identified an increased prevalence of IBD in patients with COPD and an increased risk of mortality in patients with both COPD and CD^29,30^. Furthermore, the risk of developing IBD is also increased in patients with COPD compared to healthy controls^31^. Our study showed meaningful addition to support the gut–lung axis with documentation of emphysema aggravation with microbiome dysbiosis by antibiotics and its attenuation with bacterial metabolites and postbiotics.

The benefit of antibiotics is unquestionable in modern medicine, and it is an essential treatment in COPD exacerbation caused by bacterial infection.On the other hand, antibiotic treatment represents a strong pressure that disrupts the complex microbiome ecosystem, causing an imbalance in multifaceted microbe–microbe, as well as microbe–host, interactions that are key for immune homeostasis. In this context, many previous studies have evaluated antibiotic-induced microbiome dysbiosis by antibiotics, and the majority of studies were performed in allergic and infectious disease models for respiratory diseases. Cefoperazone followed by fungal oral gavage resulted in exaggerated ovalbumin (OVA)-induced allergic airway inflammation^32^. Vancomycin treatment during pregnancy resulted in exaggerated OVA-induced allergic inflammation, characterized by higher numbers of lung eosinophils, elevated concentrations of serum OVA-specific IgE, and increased airway hyperresponsiveness. Interestingly, treatment of adult mice did not confer this effect^33^. Antibiotic mixture pretreatment significantly perturbed the composition of the intestinal microbiota and impaired host defenses against *Pseudomonas aeruginosa* pneumonia, as reflected by the increased bacterial burden and dissemination^34^. Compared to numerous studies in allergic and infectious diseases, the effect of microbiota depletion with antibiotics has not been well studied in other chronic respiratory diseases. Our study provided new evidence that microbiota depletion by antibiotics can aggravate chronic inflammatory disease and smoking-exposed emphysema. Furthermore, only treatment with the antibiotic mixture had a meaningful change in pathology, and each single antibiotic covering a specific bacterial group did not cause significant changes. This result suggested that depletion of extensive bacterial groups is necessary for emphysema aggravation compared to allergy/autoimmune diseases.

In the clinical setting of COPD, it is quite complicated to evaluate the effects of long-term and broad-spectrum antibiotics on the disease course. Broad-spectrum antibiotics are usually prescribed for repeated infections or recurrent exacerbations that cause disease deterioration^35^; thus, a causal relationship cannot be determined. Long-term macrolide or inhaled antibiotics even help to reduce exacerbations^36^, suggesting that the benefit of selective-spectrum antibiotics surpasses the potential harm of microbiota dysbiosis by eradicating causative bacteria. Considering our animal experimental data, the effort to select targeted antibiotics is necessary to preserve the microbiota and thereby ensure immune homeostasis.

The association between the intestinal microbiota and respiratory diseases has been studied. Infants with reduced abundances of *Bifidobacterium*, *Faecalibacterium*, and *Akkermansia* in the gut were noted in the highest risk group for developing asthma symptoms later in life^37^. Additionally, a lower abundance of *Akkermansia municipila* was linked to a higher severity of asthma in adults^38^. Shifts in the microbiota composition were also evident in COPD patients, and *Streptococcus* species and the *Lachnospiraceae* family also correlate with reduced lung function^39^. Smokers have a higher ratio of *Bacteroidetes* to *Prevotella* than the ratio of *Firmicutes* to *Proteobacteria* in the intestinal microbiota compared to those of nonsmokers^40,41^, and when smoking is stopped, the ratio changes at the phylum level, increasing microbial diversity and changes in the intestinal microbial composition^41^. In our study, clear separation of the intestinal microbiota with exposure to antibiotics and smoking and relative depletion of *Akkermansia municipila* and SCFA-producing bacteria, including *Lactobacillus* and *Ruminococcus*, were also noted in antibiotic-treated mice. The microbiota and their products can induce anti-inflammatory responses, and antibiotics might deplete this beneficial effect from the microbiota, although the functional significance of these changes is yet to be evaluated.

SCFAs are the first and final metabolites of dietary fiber fermentation by the intestinal microbiota, and their anti-inflammatory response has been demonstrated in various diseases, such as colitis and allergic asthma^42^. The systemic effect of the intestinal microbiota has been widely studied through SCFAs, which are also being studied to elucidate their therapeutic potential in respiratory diseases, mostly asthma models. Vancomycin exacerbates asthma and allergic lung inflammation associated with changes in the microbiome and metabolome, and propionate, a major SCFA, ameliorates this enhanced asthma susceptibility by modulating the activity of T cells and dendritic cells^43^. Feeding acetate resulted in similar results in asthmatic animal models, preferring to differentiate regulatory T cells with respect to epigenetic changes^44^. In this study, we also demonstrated the beneficial effect of SCFAs in an antibiotic-exacerbated emphysema model. SCFAs were metabolized in the liver, and their concentrations were shown to decline dramatically from the colon–cecum passage to the portal–hepatic–peripheral blood veins^45^. Considering this change, we examined three administration routes of SCFAs, and the oral and intrarectal routes showed effects corresponding to those of the intraperitoneal route, bypassing liver metabolism, in attenuating emphysema development. For clinical usage, the oral route can be applied most easily, and appropriate concentrations for this route will be a critical issue.

Autophagy is a series of processes that form autophagosomes through autophagy-related genes (ATGs) activated in response to energy loss and nutrient deficiency to maintain cell homeostasis in the body by combining with lysosomes^46,47^. Autophagy has been widely studied for its complex role in COPD^48^, and the level of the autophagy-related protein LC3B was increased in lung samples from COPD patients compared with controls^49^. Exposure to cigarette smoke extract induced the accumulation of autophagosomes and caused the elevation of LC3B protein in bronchial epithelial cell lines^50^. Autophagy has also been studied in response to antibiotics in relation to the intestinal microflora. In a study on intestinal barrier dysfunction in antibiotic-treated mice, antibiotics increased the levels of ATG5 and LC3-II/LC3-I at different time points^51^. In view of the antibiotic-induced intestinal microbiota disturbance and COPD-related autophagy, we demonstrated that antibiotics further increased autophagy in a model of exacerbated emphysema. With this result, antibiotic-induced intestinal dysbiosis can be suggested to have a role in COPD aggravation associated with autophagy.

There are some limitations to be addressed. First, the mechanism by which intestinal dysbiosis affects chronic respiratory disease should be more clearly elucidated. We found relevant changes in inflammatory markers and autophagy and the attenuation of emphysema with bacterial metabolites, but there are other mechanisms that may be related to the pathogenesis. Second, the long-term effects of antibiotics on patients with COPD should be evaluated clinically, although progressive COPD patients have a greater chance of being prescribed antibiotics, so the causal relationship may not be clear. Third, fasting and starvation can also induce autophagy in the lungs^52^; thus, the increased autophagy observed with antibiotic treatment can be affected by weight loss, which is known to be a poor prognostic factor in emphysema^53^. Emphysema aggravation and weight loss usually co-occur in both humans and animals, and they were restored together with SCFA treatment in our study. Therefore, it is not possible to separate these two factors completely. More research is needed to fully understand the complex interplay among autophagy, weight loss, emphysema, and gut dysbiosis.

In conclusion, broad-spectrum antibiotics aggravated smoking-induced emphysema with increased alveolar destruction and inflammatory factors. The gut microbiota and its metabolites are affected by smoking and antibiotics, and emphysema aggravation with antibiotics can be attenuated by microbial metabolites. Antibiotics are one of the major treatments for COPD exacerbation, and our study provides new insights into emphysema, suggesting the necessity of reasonable prescriptions of antibiotics and the therapeutic potential of restoring the gut microbiota and its metabolites.

### Supplementary information

Supplementary information

## References

[1] Centers for Disease Control and Prevention. How tobacco smoke causes disease: the biology and behavioral basis for smoking-attributable disease: a report of the surgeon general. (2010). https://www.ncbi.nlm.nih.gov/books/NBK53017/. 21452462

[2] Vogelmeier CF (2017). Global strategy for the diagnosis, management and prevention of chronic obstructive lung disease 2017 report: GOLD executive summary. Respirology.

[3] Burrows B, Knudson RJ, Cline MG, Lebowitz MD (1977). Quantitative relationships between cigarette smoking and ventilatory function. Am. Rev. Respir. Dis..

[4] Davis RM, Novotny TE (1989). The epidemiology of cigarette smoking and its impact on chronic obstructive pulmonary disease. Am. Rev. Respir. Dis..

[5] Faner R, Agusti A (2016). Multilevel, dynamic chronic obstructive pulmonary disease heterogeneity. a challenge for personalized medicine. Ann. Am. Thorac. Soc..

[6] Hooper LV, Littman DR, Macpherson AJ (2012). Interactions between the microbiota and the immune system. Science.

[7] Lloyd CM, Marsland BJ (2017). Lung homeostasis: influence of age, microbes, and the immune system. Immunity.

[8] Thavagnanam S, Fleming J, Bromley A, Shields MD, Cardwell CR (2008). A meta-analysis of the association between Caesarean section and childhood asthma. Clin. Exp. Allergy.

[9] Marsland BJ, Trompette A, Gollwitzer ES (2015). The gut-lung axis in respiratory disease. Ann. Am. Thorac. Soc..

[10] Budden KF (2017). Emerging pathogenic links between microbiota and the gut-lung axis. Nat. Rev. Microbiol..

[11] Ubags, N. D. J. & Marsland, B. J. Mechanistic insight into the function of the microbiome in lung diseases. *Eur. Respir. J*. **50**, 10.1183/13993003.02467-2016 (2017). 10.1183/13993003.02467-2016 28893867

[12] Jang YO (2020). Fecal microbial transplantation and a high fiber diet attenuates emphysema development by suppressing inflammation and apoptosis. Exp. Mol. Med..

[13] Jang YO (2021). High-fiber diets attenuate emphysema development via modulation of gut microbiota and metabolism. Sci. Rep..

[14] Dragonieri S, Carratu P, Ranieri T, Carpagnano GE, Resta O (2021). Criteria of prescription of antibiotics and systemic corticosteroids among pulmonologists and general practictioners during asthma and COPD exacerbations: a southern Italian survey. Acta Biomed..

[15] Gillespie D (2021). Associations with antibiotic prescribing for acute exacerbation of COPD in primary care: secondary analysis of a randomised controlled trial. Br. J. Gen. Pract..

[16] Schuijt TJ (2016). The gut microbiota plays a protective role in the host defence against pneumococcal pneumonia. Gut.

[17] Abt MC (2012). Commensal bacteria calibrate the activation threshold of innate antiviral immunity. Immunity.

[18] HaileMariam M (2018). S-Trap, an ultrafast sample-preparation approach for shotgun proteomics. J. Proteome Res..

[19] Bairoch A, Apweiler R (2000). The SWISS-PROT protein sequence database and its supplement TrEMBL in 2000. Nucleic Acids Res..

[20] Kall L, Canterbury JD, Weston J, Noble WS, MacCoss MJ (2007). Semi-supervised learning for peptide identification from shotgun proteomics datasets. Nat. Methods.

[21] Bolyen E (2019). Reproducible, interactive, scalable and extensible microbiome data science using QIIME 2. Nat. Biotechnol..

[22] Segata N (2011). Metagenomic biomarker discovery and explanation. Genome Biol..

[23] van Nood E (2013). Duodenal infusion of donor feces for recurrent *Clostridium difficile*. New Engl. J. Med..

[24] Trompette A (2014). Gut microbiota metabolism of dietary fiber influences allergic airway disease and hematopoiesis. Nat. Med..

[25] Ichinohe T (2011). Microbiota regulates immune defense against respiratory tract influenza A virus infection. Proc. Natl. Acad. Sci. USA.

[26] Gray, J. et al. Intestinal commensal bacteria mediate lung mucosal immunity and promote resistance of newborn mice to infection. *Sci. Transl. Med.* **9**, eaaf9412 (2017). 10.1126/scitranslmed.aaf9412 PMC5880204 28179507

[27] Bradley CP (2017). Segmented filamentous bacteria provoke lung autoimmunity by inducing gut-lung axis Th17 cells expressing dual TCRs. Cell Host Microbe.

[28] Ji XQ, Wang LX, Lu DG (2014). Pulmonary manifestations of inflammatory bowel disease. World J. Gastroenterol..

[29] Duricova D (2009). Overall and cause-specific mortality in Crohn’s disease: a meta-analysis of population-based studies. Inflamm. Bowel Dis..

[30] Vutcovici M (2016). Inflammatory bowel disease and risk of mortality in COPD. Eur. Respir. J..

[31] Ekbom A, Brandt L, Granath F, Löfdahl C-G, Egesten AJL (2008). Increased risk of both ulcerative colitis and Crohn’s disease in a population suffering from COPD. Lung.

[32] Noverr MC (2005). Development of allergic airway disease in mice following antibiotic therapy and fungal microbiota increase: role of host genetics, antigen, and interleukin-13. Infect. Immun..

[33] Russell SL (2012). Early life antibiotic‐driven changes in microbiota enhance susceptibility to allergic asthma. EMBO Rep..

[34] Wang L (2020). The microbiota protects against *Pseudomonas aeruginosa* pneumonia via γδ T cell-neutrophil axis in mice. Microbes Infect..

[35] Suissa S, Dell’Aniello S, Ernst P (2012). Long-term natural history of chronic obstructive pulmonary disease: severe exacerbations and mortality. Thorax.

[36] Wilson R, Sethi S, Anzueto A, Miravitlles M (2013). Antibiotics for treatment and prevention of exacerbations of chronic obstructive pulmonary disease. J. Infect..

[37] Fujimura KE (2016). Neonatal gut microbiota associates with childhood multisensitized atopy and T cell differentiation. Nat. Med..

[38] Michalovich, D. et al. Obesity and disease severity magnify disturbed microbiome-immune interactions in asthma patients. *Nat. Commun.* **10**, 5711 (2019). 10.1038/s41467-019-13751-9 PMC6911092 31836714

[39] Bowerman, K. L. et al. Disease-associated gut microbiome and metabolome changes in patients with chronic obstructive pulmonary disease. *Nat. Commun.* **11**, 5886 (2020). 10.1038/s41467-020-19701-0 PMC7676259 33208745

[40] Lee SH (2018). Association between cigarette smoking status and composition of gut microbiota: population-based cross-sectional study. J. Clin. Med..

[41] Benjamin JL (2012). Smokers with active Crohn’s disease have a clinically relevant dysbiosis of the gastrointestinal microbiota. Inflamm. Bowel Dis..

[42] Wypych TP, Wickramasinghe LC, Marsland BJ (2019). The influence of the microbiome on respiratory health. Nat. Immunol..

[43] Cait A (2018). Microbiome-driven allergic lung inflammation is ameliorated by short-chain fatty acids. Mucosal Immunol..

[44] Thorburn, A. N. et al. Evidence that asthma is a developmental origin disease influenced by maternal diet and bacterial metabolites. *Nat. Commun.* **6**, 7320 (2015). 10.1038/ncomms8320 26102221

[45] Cummings JH, Pomare E, Branch W, Naylor C, MacFarlane GJG (1987). Short chain fatty acids in human large intestine, portal, hepatic and venous blood. Gut.

[46] Racanelli AC, Kikkers SA, Choi AMK, Cloonan SM (2018). Autophagy and inflammation in chronic respiratory disease. Autophagy.

[47] Ornatowski W (2020). Complex interplay between autophagy and oxidative stress in the development of pulmonary disease. Redox Biol..

[48] Wu X, Yuan B, Lopez E, Bai C, Wang X (2014). Gene polymorphisms and chronic obstructive pulmonary disease. J. Cell. Mol. Med..

[49] Denardin CC (2017). Autophagy induced by purple pitanga (Eugenia uniflora L.) extract triggered a cooperative effect on inducing the hepatic stellate cell death. Cell Biol. Toxicol..

[50] Vij N, Chandramani-Shivalingappa P, Van Westphal C, Hole R, Bodas M (2018). Cigarette smoke-induced autophagy impairment accelerates lung aging, COPD-emphysema exacerbations and pathogenesis. Am. J. Physiol. Cell Physiol..

[51] Feng Y (2019). Antibiotics induced intestinal tight junction barrier dysfunction is associated with microbiota dysbiosis, activated NLRP3 inflammasome and autophagy. PLoS ONE.

[52] Nosaka N (2020). Autophagy protects against developing increased lung permeability and hypoxemia by down regulating inflammasome activity and IL-1β in LPS plus mechanical ventilation-induced acute lung injury. Front. Immunol..

[53] Marti S, Munoz X, Rios J, Morell F, Ferrer J (2006). Body weight and comorbidity predict mortality in COPD patients treated with oxygen therapy. Eur. Respir. J..

