# Supplementary material for: Systemic antibiotics cause deterioration of emphysema associated with exaggerated inflammation and autophagy
Source: Exp Mol Med. 2023 Oct 2;55(10):2260–8. doi: 10.1038/s12276-023-01099-6 (PMC10618248; doi:10.1038/s12276-023-01099-6)
Supplement: Supplementary file 1 — Supplementary information [file 12276_2023_1099_MOESM1_ESM.pdf]

Online supplement

Systemic antibiotics cause deterioration of emphysema associated with exaggerated inflammation and autophagy

Na Hyun Kim, Bo Yun Choi, Eun Sil Kim, Su jung Kim, Jeong Yeon Hong, Sun-Hee Heo, Jin-Yong Jeong, Kyunggon Kim, Hyun Ju Yoo, Woo Jun Sul and Sei Won Lee

MATERIALS and METHODS

Immunohistochemical staining

Tissues were embedded in paraffin blocks and cut into 3-µm-thick slices. The sections were placed on slides, blocked using UltraView peroxidase inhibitor (Roche Diagnostic, Indianapolis, IN,USA), and stained with antibodies diluted in Dako REAL antibody diluent (no. S2022;Agilent Technologies, Palo Alto, CA, USA). Visualization of the staining and analysis were then performed using a Ventana Benchmark XT instrument (Ventana Medical Systems). Hematoxylin stain was used for counterstaining of nuclei to produce a blue color. As a negative control, the primary antibody was omitted or replaced with an isotype control. The immunohistochemical markers used to subtype autophagy were as follows: ATG3 (1:200 [OTI2C12; Invitrogen, Carlsbad, CA, USA]), ATG5 (1:100 [PA535201; Invitrogen, Carlsbad, CA, USA]) and LC3B (1:200 [2H30L32; Invitrogen, Carlsbad, CA, USA]).

Fig S1. Comparative proteome analysis. a Venn diagram presenting the number of identified proteins in SM and SM+ABX group. **b** Principal components analysis for each group. **c** Volcano plot to compare the protein abundance between SM and SM+ABX groups. As the cut-off value of abundance > 2-fold and p < 0.5, 182 proteins were more abundant in SM group and 467 proteins in SM+ABX group. **d** Ontology process to specify the biologic process of SM+ABX group. The analysis specified autophagy and catabolic process as the dominant biologic process in SM+ABX group compared with SM group.

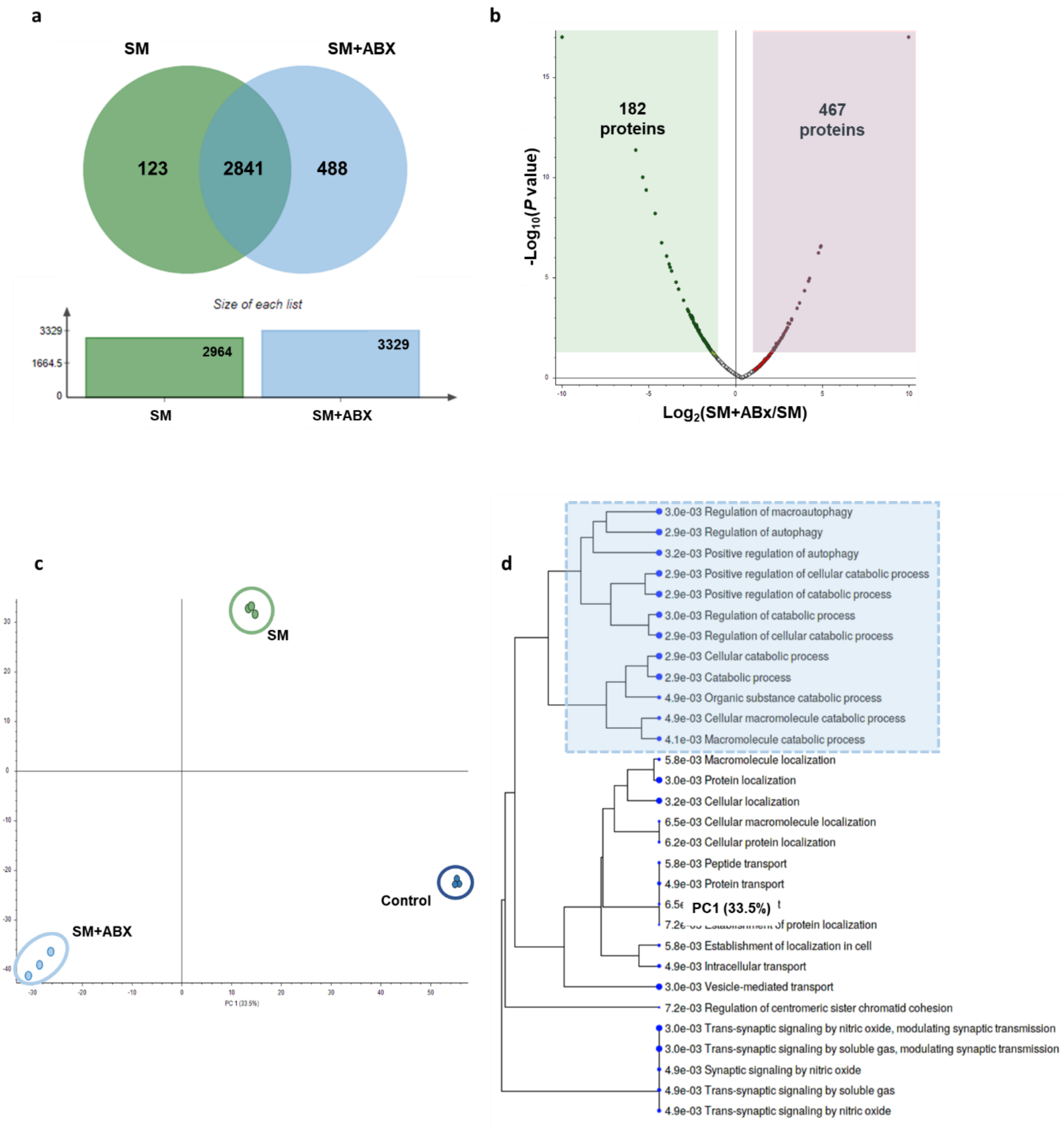

Abbreviation: SM, Smoking; SM+ABX, antibiotic mixture

Fig. S2. Immunohistochemistry staining of autophagy markers in lung pathology. **a** Immunohistochemical localization of ATG3, ATG5, and LC3B in the lungs of mice from the CTL, SM, and SMABX groups (dilution factor: ATG3, LC3B; 1:200, ATG5; 1:100). In high-resolution immunohistochemical staining, **b** ATG3, **c** ATG5, and **d** LC3B show that brown-stained alveolar epithelial cells and alveolar macrophages are darker in the SM and SMABX groups than in the CTL group (magnification: 400X, black arrow: alveolar macrophages, red triangle: pneumocyte I, red star: pneumocyte II).

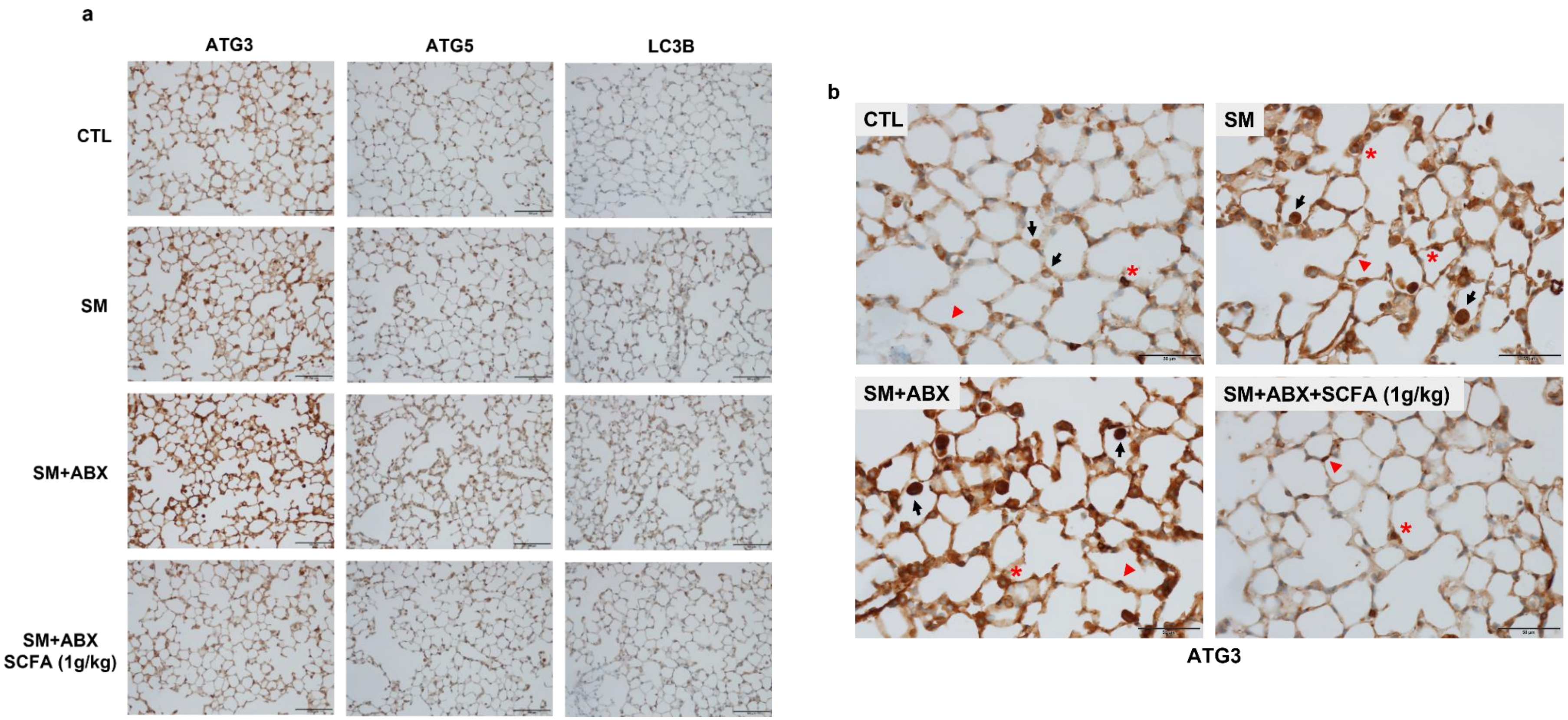

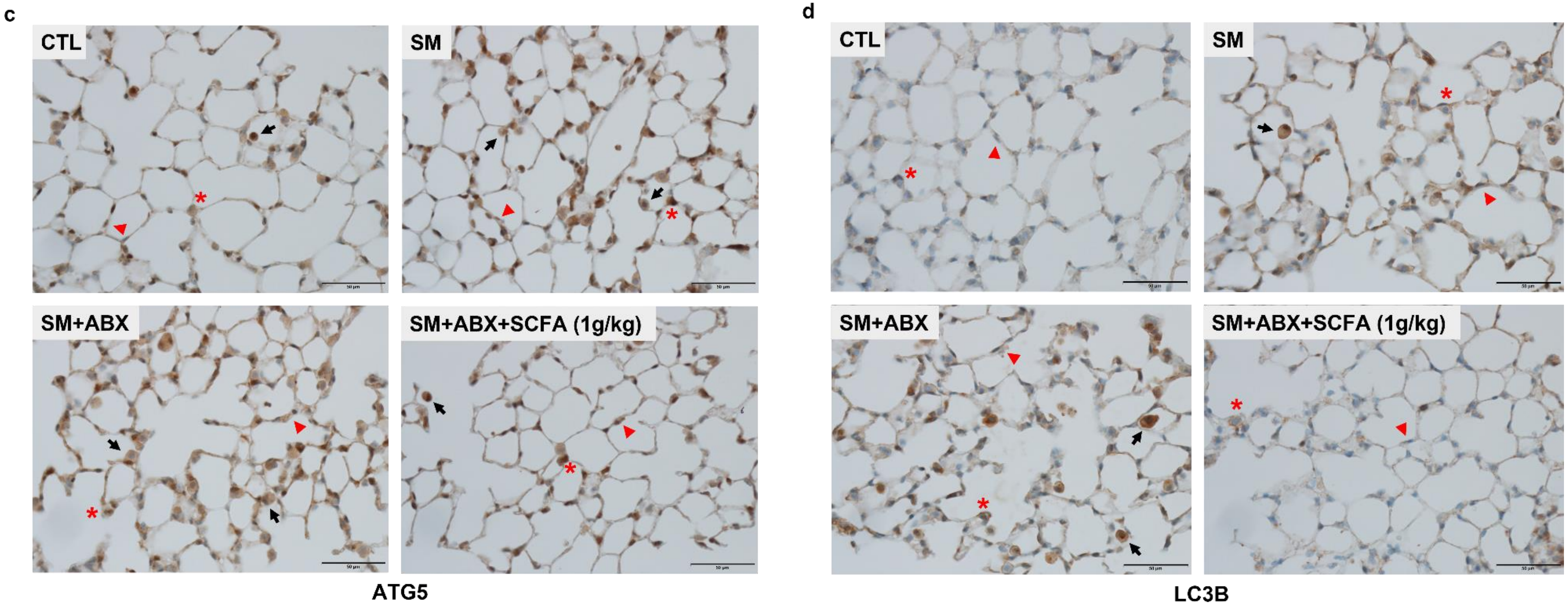

Abbreviations: CTL, control; SM, smoking; SM+ABX, antibiotic mixture; SCFA, short chain fatty acid

Fig. S3. The effect of SCFA supplementation on emphysema by concentrations. a MLI of lung tissues from each group. **b** Total number of cells in the BALF infiltrating the airways. **c** Differential cell numbers of BALF in each group. **d** Relative mRNA levels of TNFα, IL-6, and IFNγ in lung tissues.

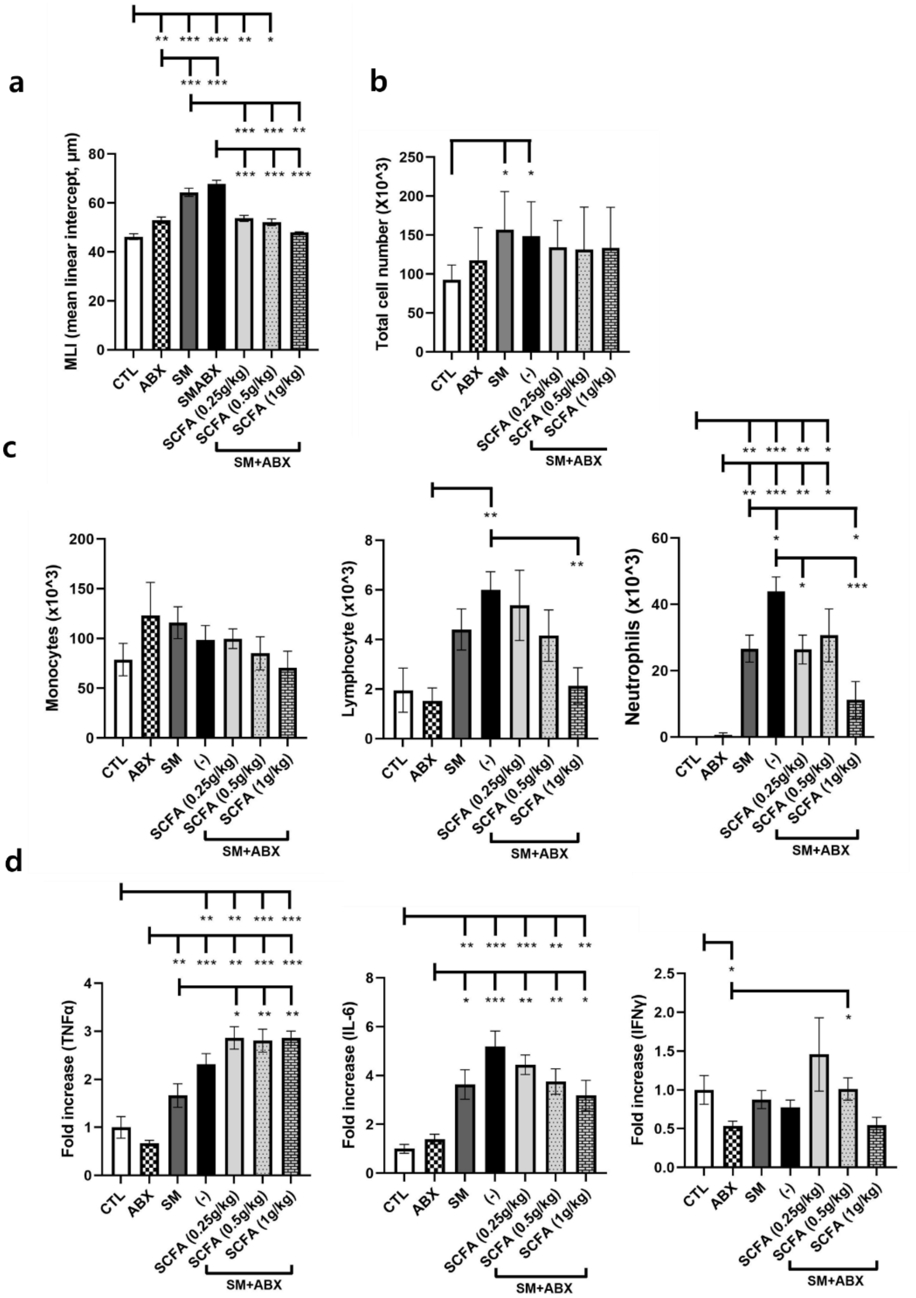

*Abbreviation*s CTL, control; ABX, only antibiotics; SM, smoking; SM+ABX, antibiotic mixture; SCFA, short chain fatty acid
